# Combined genetic screening and traditional newborn screening to improve the screening efficiency of congenital hypothyroidism

**DOI:** 10.3389/fped.2023.1185802

**Published:** 2023-05-12

**Authors:** Liang Ye, Yifan Yin, Min Chen, Nian Gong, Yong Peng, Hao Liu, Jingkun Miao

**Affiliations:** ^1^ Department of Pediatrics, Women and Children’ Hospital of Chongqing Medical University, Chongqing, China; ^2^ Department of Pediatrics, Chongqing Health Center for Women and Children, Chongqing, China

**Keywords:** congenital hypothyroidism, newborn screening, genetic screening, DUOX2, gene mutations

## Abstract

**Background:**

Congenital hypothyroidism (CH) is an neonatal endocrine disorder. Traditional newborn screening is the mainstream method of CH screening, so as to ensure the early detection and treatment of CH. This method is limited as it has high rates of false positives and negatives. Genetic screening can be used to address the shortcomings of traditional newborn Screening (NBS); however, the comprehensive clinical value of genetic screening is yet to be systematically studied.

**Methods:**

A total of 3,158 newborns who accepted the newborn screening and genetic screening were recruited for this study. Biochemical screening and genetic screening were performed at the same time. The level of TSH with the DBS was detected by time-resolved immunofluorescence assay. High-throughput sequencing technology based on targeted gene capture was used for genetic screening. The suspected neonatal was recalled and tested serum TSH, and FT4. Finally, the effectiveness of traditional NBS and combined screening was compared.

**Results:**

In this study, 16 cases were diagnosed by traditional NBS. 10 cases of *DUOX2* mutation were found in newborn CH-related genetic screening, including 5 homozygous and 5 compound heterozygous variations. We found that the c.1588A > T mutations in *DUOX2* constituting the predominant site in the present cohort.Compared with NBS and genetic screening, the sensitivity of combined screening increased by 11.1% and 55.6%, respectively. Compared with NBS and genetic screening, the negative predictive value of combined screening increased by 0.1% and 0.4%, respectively.

**Conclusions:**

Combined traditional NBS and genetic screening reduces the false negative rate of CH screening and improves the early and accurate identification of neonates with CH. Our research explains the mutation spectrum of CH in this region, and provisionally demonstrates the necessity, feasibility and significance of genetic screening in newborns and provides a solid basis for future clinical developments.

## 1. Introduction

Congenital hypothyroidism (CH) is a kind of disease which is caused by the lack of thyroid hormone synthesis and secretion due to congenital thyroid deficiency, dysplasia or defective thyroid hormone synthesis pathway, resulting in retardation of children's intellectual development and physical development (1). Children with CH have no specific clinical symptoms or mild symptoms in the neonatal period, so newborn screening (NBS) is the main method for early detection of CH. CH screening is an important part of NBS program and is widely used as a tertiary prevention intervention for birth defects. Using NBS, we can detect early asymptomatic children with CH, and timely treatment of CH is critical for optimal neurocognitive outcomes, linear growth, pubertal growth and development, and final height (2).

Traditional biochemical screening is the mainstream method of CH screening at this stage. It mainly finds the abnormal metabolic changes of children by detecting TSH (thyroid stimulating hormone) from DBSs(dried blood spots) and the levels of TSH, FT3, FT4 (free thyroxine) in serum, so as to realize the early diagnosis of the disease. However, there are several deficiencies in this test method. Firstly, the TSH level will be affected by many factors, such as gestational age, birth weight, feeding, basic diseases and so on resulting in the transient increase and false positive results (3). Secondly, special newborns at risk of CH may not produce enough TSH in the first few weeks after birth, such as premature infants and central hypothyroidism, resulting in false negative results and missed diagnosis (4, 5). Due to technical and individual differences, about 5% of children with CH cannot be detected by NBS. With the widely use of molecular diagnostic technology, several CH-related genes were discovered, such as *DUOX2, DUOXA2, TSHR,TG, TPO, SLC5A5* and so on. The development of DNA sequencing technology has transferred the focus of neonatal screening technology from metabolite level to gene level (6). At present, NGS, as a diagnostic method for children with positive or suspected NBS, is more mature in the application of genetic diseases, such as genetic metabolic diseases, severe combined immunodeficiency, and cystic fibrosis (7). In addition, BabySeq and NC-nexus sequencing suggest that target gene testing is meaningful to detect neonatal diseases, and can make up for the deficiency of traditional biochemical screening (8, 9). The ENDO-European Reference Network suggested to evaluate the etiology of CH by genetic means worldwide. It is suggested that patients with CH should undergo genetic testing to obtain more accurate diagnosis and provide the best treatment (10). The guidelines point out that any patient associated with CH syndrome should be studied from a genetic perspective to improve genetic counseling and explain this association through the discovery of new candidate genes. Therefore, we applied the combined CH screening of biochemical detection and related genes as a modified means for early detection and intervention of CH, explored the incidence of CH in some parts of Chongqing, and discussed the main mutation types and incidence rate of common CH related-genes.

## 2. Materials and methods

### 2.1. Study population and design

From January to December in 2021, a total of 3,158 neonates were included in the study and all of the parents agreed to undergo the newborn screening program and gene screening project in Women and Children' Hospital of Chongqing Medical University, including 1,590 malesand1568 females. Informed written consents were obtained from the parents of each neonate. All subjects received CH screening *via* collection of DBSs. The study design and protocol were reviewed and approved by the ethics committee of Women and Children' Hospital of Chongqing Medical University.

### 2.2. Traditional newborn screening

The methods of screening, diagnosis, and treatment were carried out according to the consensus on diagnosis and treatment of congenital hypothyroidism (10, 11). Briefly, for 7 days after 72 h of birth, the newborns were exclusively breastfed, blood was collected from the heel and dripped on special filter paper (Whatman903, China) to form DBS. Time-resolved fluoroimmunoassay (Perki-nElmer, USA) was used to measure TSH level. If the TSH level increased (9.0 mIU/L ≤ TSH < 20.0 mIU/L), the infants were recalled, and heel blood was collected for a second time and the TSH level retest. If the TSH level was ≥9.0 mIU/L, the infants were recalled again and collected their venous blood. The infant was considered normal if the second TSH level was <9.0 mIU/L. If the TSH level was ≥20.0 mIU/L, the infants were recalled, and collected their venous blood to detect the levels of serum TSH and FT4.

### 2.3. Diagnosis of CH

Venous blood from the recalled infants in the NBS was sampled to evaluate the concentrations of TSH and FT4. Serum TSH and FT4 were determined by ECL (electrochemiluminescence assay). Diagnosis of CH is based on elevated TSH levels (TSH > 4.94 mIU/L) and decreased FT4 levels (FT4 < 9.10 pmol/L). Hyper-TSH-emia (HT) was characterized by increased TSH (TSH > 4.94 mIU/L) and normal FT4 (9.10 pmol/L ≤ FT4 ≤ 19.24 pmol/L). Thyroid ultrasonography was performed to evaluate the thyroid development. The information of the diagnosed children were collected and recorded in the neonatal disease screening registration form, including birth time, gender, birth weight, gestational week and family history of thyroid disease.

### 2.4. Genomic DNA extraction and sequencing

Four blood spots with the diameter of no less than 8 mm were gathered from DBSs. Apart from that, the genomic DNA extraction system kit (QIAamp DNA Blood Midi Kit, Qiagen, Germany) was adopted, while DNA concentration was 3–25 ng/μl, and DNA purity (OD 260/280) reached 1.8–2.0. The genomic DNA is broken into small DNA fragments with a main band of 100–500 bp by Covaris LE220 ultrasonic instrument (Massachusetts, USA), and then the broken DNA fragments are screened by magnetic beads. The size of the screened main fragment is 150 bp–200 bp. The CH-related genes *DUOX2, DUOXA2, TSHR, NKX2-1, NKX2-5, FOXE1, PAX8, GLIS3, TG, TPO, SLC5A5, SLC26A4*and*IYD*were selected by a gene capture strategy, using Agilent 2100 Bio analyzer and BMG following the manufacturer's protocol. The high-throughput sequencing of the qualified enriched libraries was performed on MEGISEQ-2000 sequencer (BGI, China).

### 2.5. Analysis of data

Sequencing results were analyzed using bioinformatics methods. Spilt, comparison, and quality control were performed on the original sequencing data. We performed a search of internal databases, dbSNP, ESP6500, gnomAD, and other population databases to mark variants. Prediction software was then used to predict if the mutations were conserved and the contribution of the mutations CH pathogenesis. We searched the HGMD, PubMed, Clinvar, and other databases and literature related to the variation, and variants were analyzed following the basic criteria from American College of Medical Genetics (ACMG) guideline (12, 13). SPSS was used for ROC analysis and statistical analysis.

### 2.6. Treatment and follow-up

After diagnosis, all children were given L-T4 (the levothyroxine sodium) therapy, and recheck the thyroid function after one month of treatment, and adjust the dosage according to the test results of thyroid function, height, weight and individual differences. Under the condition of normal thyroid function, recheck once in 2–3 months within 1 year old and once in 3–4 months from 1 to 3 years old, and evaluate physical and intellectual development regularly.

## 3. Results

### 3.1. The results of traditional NBS

**Figure 1:**
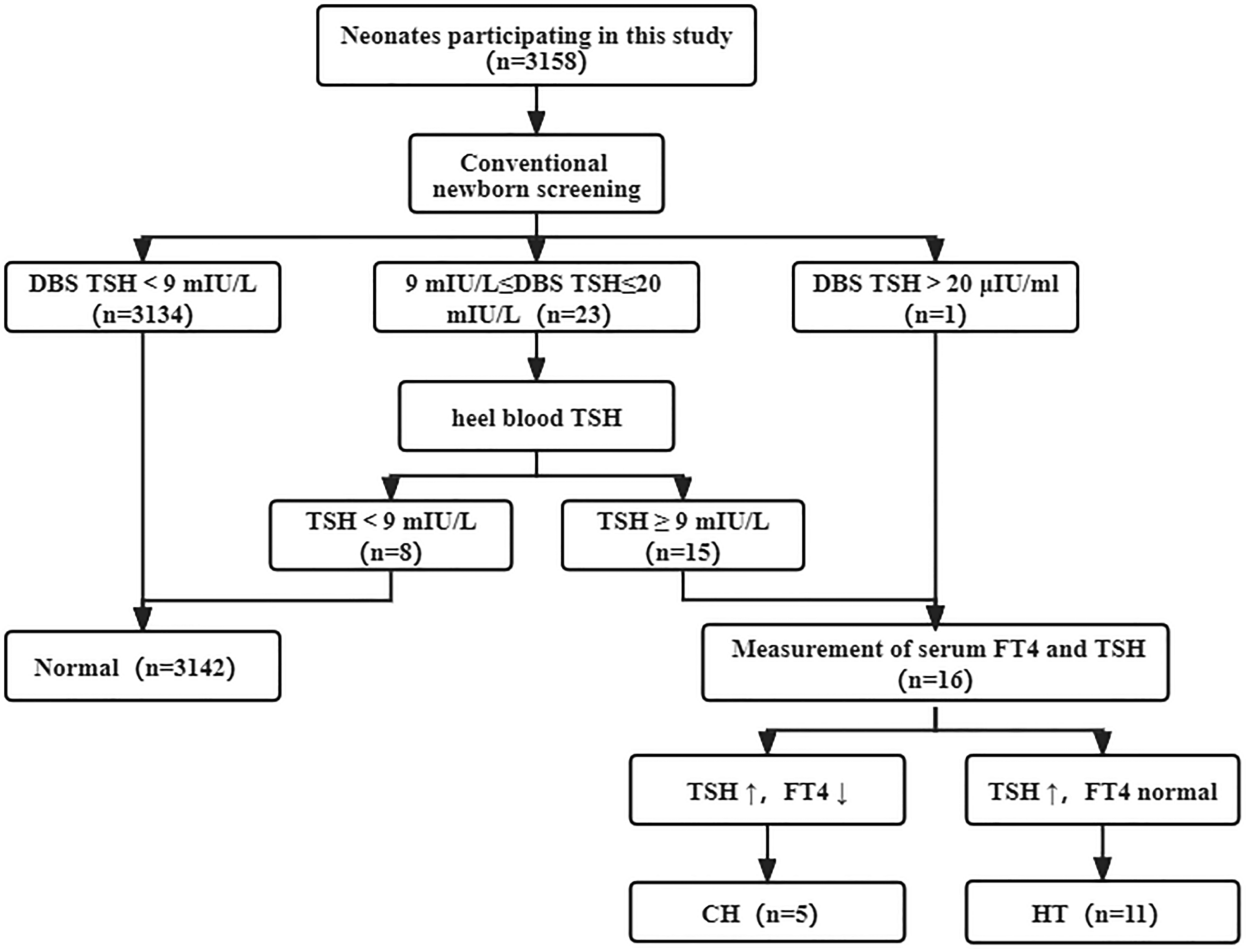
Traditional newborn screening process of CH. Of 3,158 newborns screened, 5 were diagnosed with CH and 11 were diagnosed with HT. CH, congenital hypothyroidism; HT, Hyper-TSH-emia.

Overall, 3,158 newborns were screened for CH during the study period. The screening procedure was shown in Figure 1. The initial screening results showed that 24 newborns had varying degrees of increased TSH levels.23 newborns were recalled to evaluate TSH *via* collection of heel blood. Because TSH level was more than20.0 mIU/L, one infant was recalled to detect TSH and FT4 values in venous blood. 8 of recalled newborns exhibited normal TSH levels during the recall review of heel blood. Therefore, 16 abnormal newborns were recalled to measure serum TSH and FT4 value. TSH increased in 16 abnormal newborns. FT4 decreased in 5 cases and normal in 11 cases. See Table 1 for specific screening information. All of 16 patients showed normal size/location of thyroid gland and were followed up in our center and given L-Thyroxine treatment.

**Table 1:** Biochemical characteristics of patients with CH or HT.

Patients ID	Sex	Birth weight (g)	Gestational age (week)	Heel blood TSH (mIU/L)	Thyroid morphology	Serum TSH (mIU/L)	Serum FT4 (pmol/L)	Clinical phenotype	Family history
1	Male	3,130	39	16.77	Normal	35.20	8.29	CH	No
2	Male	3,250	39	13.84	Normal	>100	5.62	CH	No
3	Male	3,030	39	13.10	Normal	48.31	8.48	CH	No
4	Female	3,250	41	46.77	Normal	88.57	6.16	CH	Yes#
5	Male	3,330	39	14.12	Normal	>100	5.51	CH	No
6	Female	3,550	39	14.21	Normal	17.92	12.79	HT	No
7	Male	3,030	40	16.56	Normal	26.21	10.19	HT	Yes#
8	Male	3,000	38	10.22	Normal	18.32	9.68	HT	No
9	Female	3,260	39	12.98	Normal	25.36	9.64	HT	No
10	Male	2,620	34	11.43	Normal	6.69	13.26	HT	No
11	Male	3,080	39	12.39	Normal	36.37	9.94	HT	No
12	Female	3,240	39	13.90	Normal	5.77	13.15	HT	No
13	Female	3,160	39	14.21	Normal	10.80	12.70	HT	No
14	Male	3,530	39	12.47	Normal	18.41	9.85	HT	No
15	Female	3,630	40	14.46	Normal	17.07	9.62	HT	No
16	Male	3,720	38	11.29	Normal	8.17	12.01	HT	No
17	Female	3,900	37	6.32	Normal	13.26	9.36	HT	No
18	Female	3,190	39	4.63	Normal	31.20	<3.86	CH	No

TSH, thyroid-stimulating hormone; FT4, free thyroxine; CH, Congenital hypothyroidism; HT, Hyper-TSH-emia.

Reference ranges for heel blood TSH, serum TSH and serum FT4 are presented. Heel blood TSH < 9.00 mIU/L; Serum TSH ≤ 4.94 mIU/L; 9.10 pmol/L ≤ serum FT4 ≤ 19.24 pmol/L.

4, #Family history of hyperthyroidism.

7, #Hypothyroidism of mother during pregnancy.

### 3.2. Results of the genetic screening

CH-related genes were detected by targeted NGS in 3,158 newborns. Based on literature review, we detected CH-related genes. In the 3,158 newborns include by the research, 10 were tested positively from *DUOX2* gene which related to thyroid dyshormonogenesis (Table 2). 8 out of the 16 patients were found with mutant genes. The other two cases were negative for NBS (Table 1). The compound heterozygous *DUOX2* mutation detected in 5 patients and the identified mutations in 5 patients are homozygous (Table 2). A total of 8 mutations sites were identified in *DUOX2* gene. The detected variants included c.1588A > T (14), c.3329G > A (15), c.2654G > T (16), c.2635G > A (17), c.3285_3286delTT, c.4537G > C, c.959T > C, and c.3516_3531delGTCCAAGCTTCCCCAG (18). We found that the c.1588A > T, c.2654G > T and c.3329G > A mutations in *DUOX2* constituting the predominant sites in the present cohort, with corresponding mutation rates of 35%, 20% and 15% (Table 3).

**Table 2:** Genetic characteristics of 8 patients with CH or HT.

Patients ID	Sex	Variants (*DUOX2*)		Clinical phenotype
1	Male	c.3516_3531delGTCCAAGCTTCCCCAG	c.2635G > A	CH
2	Male	c.1588A > T	c.1588A > T	CH
3	Male	c.4537G > C	c.959T > C	CH
4	Female	c.4537G > C	c.2654G > T	CH
6	Female	c.3329G > A	c.3329G > A	HT
7	Male	c.2654G > T	c.2654G > T	HT
14	Male	c.1588A > T	c.1588A > T	HT
15	Female	c.1588A > T	c.1588A > T	HT
17	Female	c.3329G > A	c.1588A > T	HT
18	Female	c.3285_3286delTT	c.2654G > T	CH

CH, Congenital hypothyroidism; HT, Hyper-TSH-emia.

**Table 3:** Potential pathological *DUOX2* variants detected in the present study.

Location	cDNA change	Amino Acids change	ACMG classification	Mutation type	No. of cases	Frequency (%)
Exon 14	c.1588A > T	p.K530*	P	Nonsense	7	35
Exon 20	c.2654G > T	p.R885l	LP	Missense	4	20
Exon 25	c.3329G > A	p.R1110Q	P	Missense	3	15
Exon34	c.4537G > C	p.G1513R	P	Missense	2	10
Exon 9	c.959T > C	p.L320P	VUS	Missense	1	5
Exon 10	c.2635G > A	p.E879K	P	Missense	1	5
Exon 27	c.3516_3531delGTCCAAGCTTCCCCAG	p.Lys1174Serfs*12	LP	Frameshift	1	5
Exon 25	c.3285_3286delTT	p.Ile1097Leufs*24	LP	Frameshift	1	5

P, pathogenic; LP, likely pathogenic; VUS, uncertain significance.

*termination codon.

### 3.3. Comparison of traditional NBS and genetic screening results

We then compared positive results from traditional NBS and genetic screening. Among them, 16 patients were diagnosed by traditional NBS, and 10 children were diagnosed by genetic screening. It is noteworthy that 18 patients were found by combined screening (Table 1). The NBS of two newborns were normal, and TSH was 4.63 mIU/L and 6.32 mIU/L, respectively (Table 1). The TSH value was within the normal range. But genetic screening revealed *DUOX2* mutations. The genotypes were compound heterozygous mutation c.3329G > A/c.1588A > T and c.3285_3286delTT/c.2654G > T, respectively. The pathogenicity of the above mutation sites is clear, and cases have been reported in many literatures (18). Therefore, we recalled them for thyroid function test. Patient 17 revealed that TSH level was 13.26 mIU/L and FT4 was normal, showing HT. Patient 18 revealed that TSH level was 31.20 mIU/L and FT4 was decreased, showing CH. Genetic Screening played an important role in helping the timely diagnosis and treatment of the above two patients.

**Figure 2:**
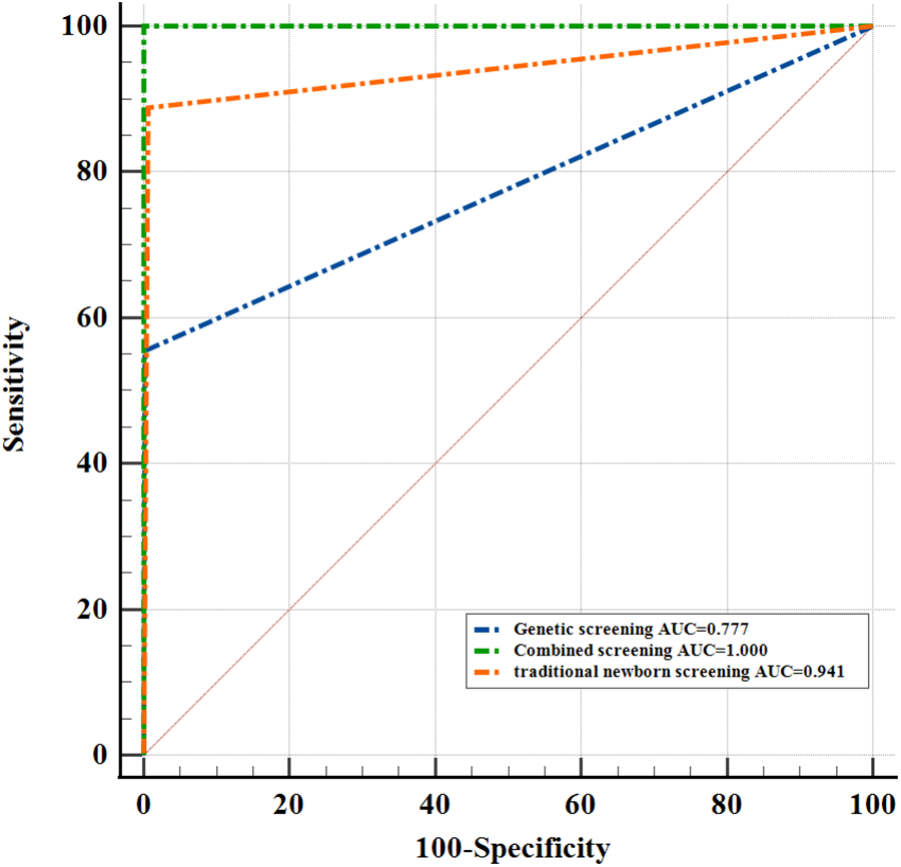
Comparison of ROC curves of traditional newborn screening, genetic screening and combined screening.

With clinical diagnosis as the reference standard, the sensitivity of traditional NBS and genetic screening was 88.9% and 55.6%, respectively. But the sensitivity of combined screening was 100% (Table 4). Compared with traditional NBS and genetic screening, the sensitivity of combined screening increased by 11.1% and 44.4%, respectively. Combined screening improves the sensitivity of screening and reduces the false negative rate. The specificity of traditional NBS, genetic screening and combined screening was 100%. In addition, the positive and negative predictive value of traditional screening were 100% and 99.9%. The positive and negative predictive value of genetic screening were 100% and 99.7%. Compared with traditional NBS and genetic screening, the negative predictive value of combined screening increased by 0.1% and 0.3%, respectively. We found that the AUC of traditional NBSwas0.941, the AUC of genetic screening was0.777, and the AUC of combined screening was1.000 (Figure 2). The results suggested that children with CH who were missed by biochemical tests were found through genetic screening, and combined screening reduced the false-negative rate of CH screening. Genetic screening could be used as a supplementary diagnostic method for traditional NBS.

**Table 4:** Diagnostic results.

		NBS		Genetic screening		Total
		+	−	+	−	
Genetic screening with NBS	+	16	2	10	8	18
	−	0	3,140	0	3,140	3,140
	Total	16	3,142	10	3,148	3,158

+, positive screening; −, negative screening; NBS, newborn screening.

### 3.4. Clinical features of 18 cases

A total of 3,158 neonates were screened in this project, including 94 premature infants, with a preterm birth rate of 2.98%. Among 18 patients, 6 cases of CH were diagnosed with an incidence of 1/526, 12 cases of HT were diagnosed with an incidence of 1/263. The overall incidence of hypothyroidism was 1/175. Among them, 10 were males and 8 were females (Table 5). The median birth weight of our research was 3,245 g.The median gestational week was 39 weeks, and one was premature baby. Among the 18 children, 17 were AGA, 1 was LGA, and no SGA. 2 of 18 cases had family history of thyroid disease. The results showed that there were no obvious abnormalities on thyroid development and morphology of all 18 children. There had no abnormal condition at birth.

**Table 5:** Clinical features of the 18 cases.

*n* (males/females)	18 (10/8)
Birth weight, median (IQR) (g)	3,245 (3,093–3,480)
Gestational week, median (IQR) (week)	39 (39–39)
**Relationship between birth weight and gestational age (*n*)**	
SGA	0
AGA	17
LGA	1
**Mode of delivery (*n*)**	
Natural childbirth	11
Assistant delivery	7
Family history of thyroid disease(*n*)	2
**Thyroid morphology (*n*)**	
Normal	18
Abnormal	0
Abnormal at birth (dystocia, premature rupture of membranes, asphyxia) (n)	0

CH, Congenital hypothyroidism; HT, Hyper-TSH-emia; IQR, interquartile range; SGA, small for gestational age; AGA, appropriate for gestational age; LGA, large for gestational age.

## 4. Discussion

CH is an endocrine disease caused by hypothyroidism due to insufficient thyroid hormone synthesis and secretion. It is one of the main items of newborn disease screening in the world (19). Researches show that the incidence of CH is 1/2,000–1/4,000 (20), while the incidence in China is about 1/2,400, and there are obvious regional differences (1, 21).Our research shows the incidence of CH was 1/526 and incidence of HT was1/263. The prevalence of CH in Chongqing was higher than the average prevalence of China. One factor contributing to this change may be increased screening of newborns at higher risk of congenital hypothyroidism, including premature infants (22) and particular ethnic populations with a family history of thyroid disease. Methodological improvements in newborn screening appear to be another factor in the rising incidence of CH, specifically the optimization of TSH screening cut-offs and using genetic screening methods. Therefore, the incidence of HT is increased, which indicates that the incidence of transient CH is increased.

NBS for CH is performed routinely in most regions of China, where it has led to decrease intellectual disability caused by this common condition. Early detection is important as treatment should be initiated as early as possible, preferably within the first two weeks of life. Newborn children usually have an improved prognosis following early L-T4 therapy. However, NBS for CH is loaded with a high rate of false-negative results, which appears to be inevitable because the TSH and FT4 concentration at birth is easily affected by maternal and other factors. These factors include premature, low birth weight, and central hypothyroidism (4, 5). Furthermore, some research indicated that false positives can cause newborns and their families to be recalled to the hospital for reexamination which may take a long time. This process will also increase family anxiety. Additionally, newborns at risk of CH may not produce enough TSH in the first few weeks after birth, and the screening results may be false negative at this time. It was recommended that the second screening and follow-up of twins should be carried out 2 weeks after birth or 2 weeks after the first screening by guidelines In our study, NBS results found 11 of the children with HT. And four of them had *DUOX2* mutation. More important, genetic screening was performed on 3,158 neonates using targeted next-generation sequencing, and two CH cases of false-negative were identified. Therefore, combining traditional NBS with genetic testing is crucial to improve screening sensitivity.

According to previous reports, the cause of CH in approximately 80%–85% of patients is thyroid dysgenesis (including agenesis, ectopy, and hypoplasia), which is related to gene mutations in *TSHR*, *PAX8*, *TTF1/NKX2-1*, and *TTF2/FOXE1*. Interestingly, all CH children had euthyroid glands and no thyroid dysplasia. And there was no *TSHR* gene mutation in this study. In addition, 10%–15% of cases are caused by thyroid dyshormonogenesis, which is associated with mutations in thyroid *DUOX2*, *DUOXA2*, *TG*, and *TPO* (23, 24). These genes play important roles in the growth and development of the thyroid gland. Reports on CH caused by the *DUOX2*, *DUOXA2,* and *TSHR* gene have been gradually increasing (18). This study revealed the *DUOX2* variant spectrum in some population of Chongqing, China. In our study, only the *DUOX2* variant was found. Compared with *DUOXA2* and *TSHR*, *DUOX2* variants are more prevalent in the Chongqing population. According to previous studies, mutations in *DUOX2* are responsible for thyroid dyshormonogenesis (25). Several studies have suggested that the most reported variants among Chinese, Japanese, and Thai patients with CH have been identified in *DUOX2*, suggesting that *DUOX2* variants are an even more frequent causative factor for CH than previously recognized. Previous studies have shown that the detection rate of *DUOX2* mutations in children with CH in China is as high as 28%–44%whereas the detection rate obtained in this study was 44% (8/18), suggesting that *DUOX2* mutations may be the main cause of CH in the population of Chongqing (26). In particular, the c.1588A > T and c.3329G > A variants have been reported in Asian populations, including Chinese (27–29), Japanese (30), and Korean (31), patients. And c.2654G > T (32) mutations are also predominant in Asians, mostly in the Chinese Han population. The c.1588A > T and c.3329G > A variants in *DUOX2* were also identified as the most frequent sites in this study. In our research, some children failed to detect mutations, so the range of CH-related genes should be expanded in subsequent studies to find more mutation sites.

Compared with traditional NBS, combined genetic screening and traditional NBS applied to CH screening could be drive down the cost of healthcare by reducing, or possibly eliminating, unnecessary patient follow-up and laboratory testing. And early acquisition of the genotype of the neonates is beneficial for the precise diagnosis, treatment, and prevention and control of CH (33, 34). This is very important for early treatment of newborns, and improving the psychological and physical development of newborns. This is also necessary for the current consensus guidelines on CH (10). However, several limitations should be considered in the study when reviewing our findings. First, the sample size is relatively small; studies based on a larger cohort need to be conducted in the future to confirm our findings. Secondly, reasonably select CH-related genes and consider the cost-effectiveness of combined screening. It is necessary to fully consider the genetic characteristics such as variation spectrum and incidence rate of population in advance.

This study conducted a preliminary assessment of the conventional screening and genetic screening methods of CH, so as to provide a basis for the research of newborn genetic screening in China. Incorporating genetic screening and traditional biochemical screening into neonatal screening is conducive to expanding the scope of disease screening, avoiding missed detection and reducing the false positive rate. However, the genetic screening strategies for many rare diseases are still in the exploratory stage, and the genes and loci covered by newborn CH genetic screening in China need to be further evaluated. Overall, our research has temporarily proved the necessity, feasibility and significance of neonatal clinical gene screening. The combination of CH genetic screening and traditional biochemical screening can maximize the role of NBS, but more research is needed to further improve it.

## Data Availability

The original contributions presented in the study are publicly available. This data can be found here: https://figshare.com/s/c806c6753e34a072d60f.
